# Development of a high-throughput minimum inhibitory concentration (HT-MIC) testing workflow

**DOI:** 10.3389/fmicb.2023.1079033

**Published:** 2023-05-25

**Authors:** Suman Tiwari, Oliver Nizet, Nicholas Dillon

**Affiliations:** ^1^Department of Biological Sciences, University of Texas at Dallas, Richardson, TX, United States; ^2^La Jolla Country Day School, La Jolla, CA, United States

**Keywords:** Opentrons, MIC, OT-2, high-throughput, Python, robot, antibiotic

## Abstract

The roots of the minimum inhibitory concentration (MIC) determination go back to the early 1900s. Since then, the test has undergone modifications and advancements in an effort to increase its dependability and accuracy. Although biological investigations use an ever-increasing number of samples, complicated processes and human error sometimes result in poor data quality, which makes it challenging to replicate scientific conclusions. Automating manual steps using protocols decipherable by machine can ease procedural difficulties. Originally relying on manual pipetting and human vision to determine the results, modern broth dilution MIC testing procedures have incorporated microplate readers to enhance sample analysis. However, current MIC testing procedures are unable to simultaneously evaluate a large number of samples efficiently. Here, we have created a proof-of-concept workflow using the Opentrons OT-2 robot to enable high-throughput MIC testing. We have further optimized the analysis by incorporating Python programming for MIC assignment to streamline the automation. In this workflow, we performed MIC tests on four different strains, three replicates per strain, and analyzed a total of 1,152 wells. Comparing our workflow to a conventional plate MIC procedure, we find that the HT-MIC method is 800% faster while simultaneously boasting a 100% accuracy. Our high-throughput MIC workflow can be adapted in both academic and clinical settings since it is faster, more efficient, and as accurate than many conventional methods.

## Introduction

Alexander Fleming first reported the inhibitory effect of antibiotics against staphylococci in 1929 by serially diluting penicillin in nutrient broth. He observed that the opacity of the broth, or the culture’s pH, could be used to detect the inhibition of a bacterial suspension (Fleming, 2001; Wheat, 2001). Through expanding this technique, he made it possible to gauge the inhibitory potency of antibiotics against various bacterial species. Fleming’s early work has been described as a forerunner of contemporary minimum inhibitory concentration (MIC) methodology. A MIC is defined as the lowest concentration of an antibacterial agent expressed in mg/mL (g/L) which, under strictly controlled *in vitro* conditions, prevents visible growth of the test strain (Kowalska-Krochmal and Dudek-Wicher, 2021). Although an *in vitro* MIC does not always indicate *in vivo* bacterial susceptibility, they are still the most common and best predicter of clinical antibiotic efficacy available (Reller et al., 2009; Kowalska-Krochmal and Dudek-Wicher, 2021).

Antibiotic susceptibility testing using standardized disk diffusion was introduced by Bauer and Kirby’s experiments in 1956. In this method, the isolated bacterial colony is selected, suspended into growth media, and standardized through a turbidity test. The standardized bacterial suspension is inoculated onto a solidified agar plate and an antibiotic-treated paper disk is added. The antibiotic on the disc permeates through the agar and forms a zone of inhibition where bacteria do not survive (Khan et al., 2019). Whilst antimicrobial activity methods based on agar diffusion have clear benefits in terms of efficiency and cost, there are drawbacks, and the validity and appropriateness of agar diffusion methods for accurately quantifying antimicrobial activity has been questioned (Bonev et al., 2008; Eloff, 2019). With the advancement of technology, MIC values were later determined by broth dilution using a microplate reader to reduce the dependence on human observation.

Microplate-readers are instruments designed to measure the absorbance, fluorescence, or luminescence of samples in microtiter plates and are used for a variety of applications (Thompson, 2022). The microtiter plates, first introduced in the 1970s, can be utilized to conduct MIC experiments in low volumes (Ashour et al., 1987). Their primary benefits include low sample volumes, high experimental throughput, and simplicity of usage.

In the 1980s the Clinical and Laboratory Standards Institute (CLSI) consolidated the methods and standards for MIC determination for clinical usage (Wikler, 2006). Large sets of MIC sampling, including multiple strains and antibiotics, are time-consuming using the standardized approaches. We require new high-throughput sample handling methods as big data usage is becoming more commonplace. Recent developments in information technology and robotics have optimized manual labor operations and could now be used to modernize and streamline MIC testing (Holland and Davies, 2020).

We have developed a high-throughput MIC (HT-MIC) testing workflow to streamline and enhance previous standardized approaches as described by CLSI in “Methods for Dilution Antimicrobial Susceptibility Tests for Bacteria That Grow Aerobically, 11th Edition” (CLSI, 2018). Notably, as compared to the methods described in the “Methods for Dilution Antimicrobial Susceptibility Tests for Bacteria That Grow Aerobically, 11th Edition,” the HT-MIC workflow allows for a more precise inoculation culture, quantitatively determines antibiotic inhibition, reduces dilution error, and is adapted for high-throughput applications using a multi-step arraying system paired with robotics. The HT-MIC workflow uses an Opentrons OT-2 robot (Opentrons Inc., New York, NY, USA), a benchtop liquid handling tool that can transfer liquids using disposable tips and electronic pipettes (Tenhaef et al., 2021). The OT-2 is one of many automated liquid handling systems now on the market, but it stands out due to its low cost, which should make it feasible for both research and clinical use (Liang et al., 2021). Also, the Opentron robot’s glass barrier restricts airflow across samples leading to an even lower rate of contamination than benchtop pipetting (Opentrons, 2019). A wide variety of industrial processes have seen improvements in their production rates, efficiency, and quality thanks to the gradual incorporation of automation into workflows (Hitomi, 1994). The Opentrons OT-2 robot is used to generate master and testing plates, growth inhibition is measured on a microplate reader, and Python programming assigns MIC values free of human error. We propose the HT-MIC method is faster, more efficient, and more accurate than conventional methods.

## Materials and equipment

### Opentrons robot

The workflow mentioned in this paper utilizes the Opentrons OT-2 robot with a removable P300 multi-channel Gen1 pipette mounted on the right side and a P50 single-channel Gen1 pipette (not used in the protocol) mounted on the left side of the robot.

### Medium

Cation Adjusted Mueller-Hinton broth (CA-MHB) is CLSI approved media and is used as a medium for cultures and broth dilution in this workflow. In general, it has few antagonists and promotes healthy growth of the majority of non-fastidious pathogens [European Committee for Antimicrobial Susceptibility Testing (EUCAST) of the European Society of Clinical Microbiology and Infectious Diseases (ESCMID), 2003].

### Bacterial strains, antibiotics, and other materials

*Acinetobacter baumannii* strains AB ATCC 19606, BAA 1605, BAA 1710, and BAA 1789 were obtained from the American Type Culture Collection (ATCC) and stored at −80°C in 20% glycerol (Biobasic) and 80% CA-MHB (Difco) (Dillon et al., 2019).

**FIGURE 1:**
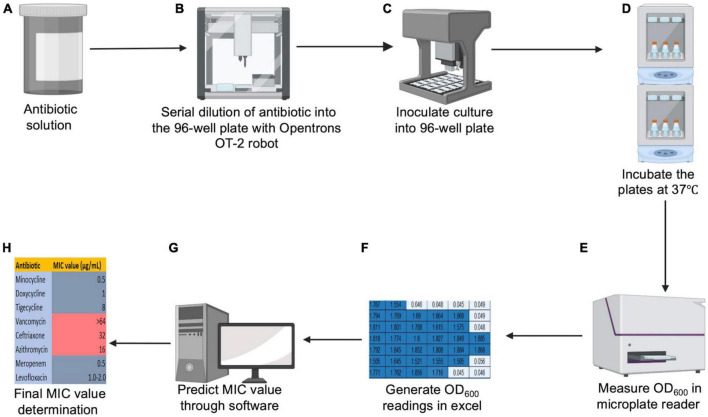
Schematic diagram for HT-MIC. **(A,B)** Stock concentrated antibiotic solutions are made and serially diluted into the 96 well plate using OT-2 robot. **(C)** Bacterial cultures are grown and 96 well plates are inoculated using the OT-2 robot. **(D,E)** Plates are incubated overnight in the orbital shaker, and the OD_600_ values are measured in a microplate reader. **(F–H)** The generated excel sheet of OD_600_ readings is loaded into the prediction software to predict final MIC value.

Concentrated stocks of azithromycin (Fresenius kabi, Bad homburg, Germany) and ciprofloxacin (Sigma, St. Louis, Missouri, MO, USA) were prepared at 100 mg/mL in DI H_2_O. Doxycycline (Fresenius kabi, Bad homburg, Germany), vancomycin (Mylan, Canonsburg, Pennysylvania, PA, USA), and meropenem (Hospira, Lake Forest, Illinois, IL, USA) were prepared at 50 mg/mL in DI H_2_O. Minocycline (Melinta, Parsippany-Troy Hills, New Jergey, NJ, USA) at 20 mg/mL, tigecycline (Fresenius kabi, Bad homburg, Germany) at 0.45 mg/mL, and levofloxacin (Sigma, St. Louis, Missouri, MO, USA) at 1.44 mg/mL were all also prepared in DI H_2_O. Fresh 100 × antibiotic experimental stocks were made at the desired concentration prior to the start of each experiment (Figure 1A). The cultures were grown in an orbital shaker (Innova S44), and the OD_600_ was measured using a plate reader (Synergy H1).

## Methods

### Opentrons MIC testing workflow

**FIGURE 2:**
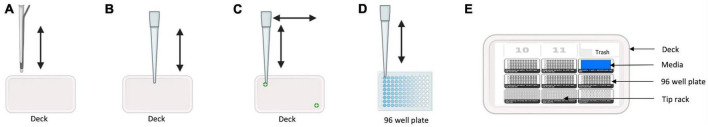
Calibration steps illustration. **(A)** Unloaded pipette moves vertically to determine pipette height from the deck. **(B)** Tip loaded pipette moves up and down to determine the distance from the deck. **(C)** Tip loaded pipette moves vertical and horizontal to reference points engraved in the robot’s deck to record its motor movement between the points. **(D)** Tip loaded pipette moves to the top of well A1 to determine if centered. **(E)** Labware position check for tip racks, 96 well plates, media reservoir, etc., to determine correct position of each labware.

Figure 1 illustrates the workflow for our HT-MIC testing method using multiple modules on the Opentrons OT-2 robot. The protocol for the use of the OT-2 robot was created using the Opentrons protocol designer beta program. Before using, the Opentrons robotic platform needs to be calibrated to manually put the liquid-handling arm in the predetermined position for each slot (such as 96-well plates, a tip box, the garbage, etc.) (Opentrons, 2019). The calibration steps include tip length calibration, pipette offset calibration, deck calibration, and labware position check (Figure 2). The steps are performed using Opentrons software and the application will guide the operator through the required steps for calibration. For the tip length calibration and pipette offset calibration, the unloaded pipette will be manually adjusted to determine the pipettes height from the deck using Opentrons application cursors (Figure 2A). A tip will be then captured by the pipette, and the distance of the loaded pipette from the deck will be determined (Figure 2B). The OT-2 stores this information for the particular pipette and model of tip for use in future applications. Then, the pipette will be moved to a predetermined reference point by the OT-2. The pipette will be manually centered over the well using the Opentrons application. In the deck calibration step, the Opentrons application will display reference points that have been precisely engraved into the deck surface when you calibrate the deck. Each of those locations will be manually entered using the pipette by jogging the cursor in the Opentrons application (Figure 2C). This demonstrates to your OT-2 how to operate its motors to move between those points. The pipette tip will be moved by the OT-2 to the top of well A1 (Figure 2D). If necessary, the pipette has to be manually adjusted using the Opentrons application so that it is properly centered at the top of well A1. That modification will be saved for when the OT-2 executes a protocol. Finally, the projected position of a specific arrangement of the labware definition, deck slot, and OT-2 is fine-tuned by the labware position check (Figure 2E).

**FIGURE 3:**
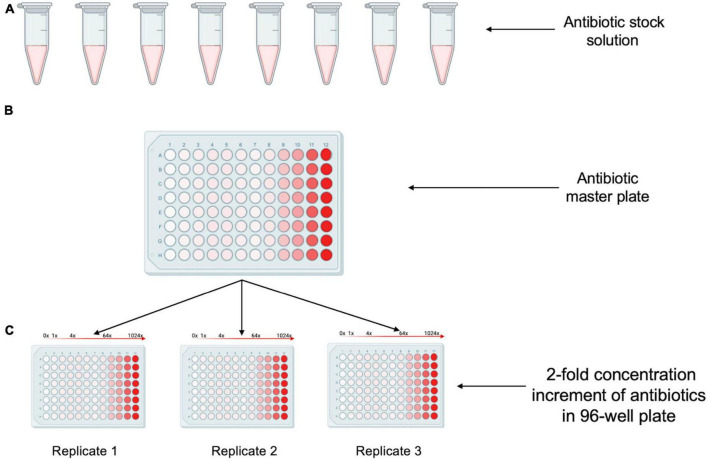
Creation of antibiotic stock plates. **(A)** Desired concentrated antibiotics stock solutions are made in an microcentrifuge tube. **(B)** The antibiotic solutions are dispensed into a 96-well plate containing media to create the master plate. **(C)** Multiple antibiotic stock plates are generated by dispensing the contents from the master plate to new 96-well plates.

**FIGURE 4:**
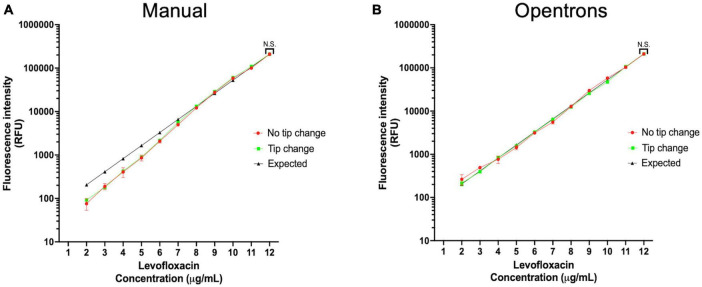
Serial dilution accuracy is not influenced by changing pipette tips. **(A)** Frequency of pipette tips change and no tip change in manual pipetting does not affect the dilution factor. **(B)** Frequency of pipette tips change and no tip change in robotic pipetting does not affect the dilution factor.

The workflow begins with the pre-processing module to generate an antibiotic master plate containing serially diluted working stocks of each antibiotic. In the first step of this module CA-MHB, or the desired diluent for the antibiotic, is added to each well of columns 1–11 (150 μL) and to column 12 (270 μL) to facilitate serial dilutions. Each 100× antibiotic stock is then added to column 12 of the 96-well plate through diluting 30 μL of the 100× stock into 270 μL of media to create a final 10× working stock. Well contents are mixed, and the pre-processing module pipettes 150 μL from column 12 to column 11 to start the twofold dilution scheme. Twofold serial dilutions continue from column 12 until column 2 with the 300 μL pipette tips (Opentrons) discarded and reloaded after dispensing into column 9, column 6, and Column 2. Using this layout scheme each row contains 11 concentrations of each antibiotic arrayed in twofold increments. Antibiotics are intentionally omitted from column 1 to create a no-drug control (Figure 3). Using the inherent fluorescence of levofloxacin (excitation 340 nm and emission 410 nm) we experimentally tested the necessity of changing pipette tips while doing serial dilutions manually or on the Opentrons system. We calculated an expected value for each well based on serial two-fold dilutions of the highest concentration well. Tips were either changed between every well, or not changed throughout the entire dilution scheme (11 total dilutions). Measured values were not significantly different from expected values, indicating the same tips can be used during the serial dilutions to reduce tip waste if desired (Figure 4).

**FIGURE 5:**
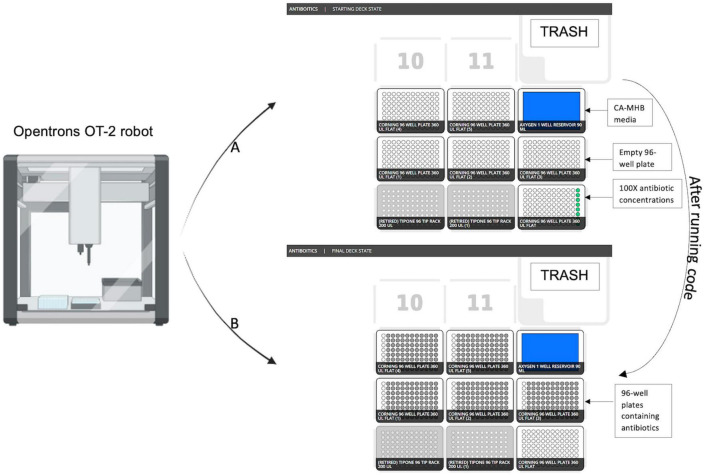
Opentrons OT-2 robot initial and final deck state after running protocol. **(A)** Starting deck state before running the protocol. **(B)** Final deck state after running protocol.

In the next step, the antibiotic master plate is used to create 5 identical MIC test plates. The Opentrons deck state for five 96-well MIC test plates is shown utilizing 300 μL pipette tips (Opentrons) in deck 1 and 2, the master plate in deck 3, the test plates in decks 4–8 (Figure 5) and 90 mL well reservoir (Axygen) in deck 9.30 μl of antibiotic solution from each well of the master plate is replica plated into each of the five 96-well MIC test plates. As the antibiotic can be diluted in either the desired media, or in a different diluent of choice, optimal drug solubility is maintained during stock plate creation. These steps can be repeated multiple times using the protocol until the desired quantity of 96-well plates is made. The code for the pre-process module for the Opentrons OT-2 robot is provided in the GitHub repository under “antibiotics.json” file name. JavaScript Object Notation (JSON) documents are both machine- and human-readable, and storing the metadata in the JSON format preserves the adaptability of the created metadata with various research data management tools and processes (Chaerony Siffa et al., 2022).

### MIC testing

Each independent replicate culture was grown overnight at 37°C shaking on an orbital shaker (Innova S44) at 200 rpm in CA-MHB (+10 mg/L MgCl_2_ (Fisker) and 20 mg/L CaCl_2_ (Sigma, St. Louis, Missouri, MO, USA) to stationary phase. The optical density (OD) of a bacterial cell culture can be determined spectrophotometrically, which is a practical and widely used approach to assess its growth stage (Meyers et al., 2018). Fifty μL of overnight cultures was inoculated into fresh CA-MHB the day of the experiment and grown to mid-log phase (OD_600_ = ∼0.4, ∼1*10^8^ CFU/mL) at 37°C and 200 rpm (Dillon et al., 2019). To measure mid-log phase of cultures at OD_600_, 300 μL of culture is pipetted into a 96 well plate (Costar) and measured on a 96-well plate reader (Synergy H1) with a 5s shaking step prior to the measurement and CA-MHB used as a blank. Mid-log phase cultures were diluted to ∼ 1*10^5^ CFU/mL (OD_600_ = ∼0.002) and 270 μL of cultures were added to 96-well (Costar) plates that contained 30 μL antibiotics dispensed using our HT-MIC Opentrons workflow protocol (Figure 1C) provided in the GitHub repository under “MIC test.json” file name. In this protocol, there are 300 μL pipette tips (Opentrons) in deck 1 and 2, 90 mL well reservoir (Axygen) containing cultures in deck 3, and stock antibiotic plates in deck 4 and 5. Plates are covered with their lids to prevent evaporation and the plates are placed into a ambient air orbital shaking incubator and grown for approximately 20 h shaking at 200 rpm at 37°C (CLSI, 2018; Dillon et al., 2019; Salazar et al., 2020; Figure 1D). This setup is designed for MIC determination at 20 h, if longer incubations are desired then incorporation of breathable membrane covers for the 96-well plates can be utilized to prevent evaporation. After incubation, the 96-well plates were removed and the OD_600_ of each well was measured on a 96-well plate reader (Synergy H1) with a 5 sec shaking step prior to the measurement and CA-MHB used as a blank (Figure 1E). Measured OD_600_ values were exported into excel for MIC determination (Figure 1F). This protocol is used to determine high confidence MIC_90_ values. The MIC_90_ is defined as the lowest concentration of an antibiotic that inhibits ≥ 10% of the bacterial growth found in the no-antibiotic control (Liang et al., 2019). Growth conditions, and MIC experimental duration is optimized for our pathogens of interest, but media, culture densities, and incubation conditions can be adjusted as needed.

### Automated MIC determination

The automated MIC determination is accomplished using a Python program provided in GitHub repository under “MIC-PythonCode.docx” file name. The program input is an Excel file comprised of two sheets provided in GitHub repository under “example input data.xlsx” file name. The first sheet includes the measured OD_600_ values for three different 96-well plates, each sharing the same antibiotics and concentration ranges. Each row in the spreadsheet corresponds to one of the eight antibiotics, and the 36 columns correspond to the OD_600_ readings repeated in triplicate for each of 12 concentrations. The second sheet possesses metadata detailing the concentrations of antibiotics corresponding to each of the OD_600_ values on the first sheet. The program first reads the Excel file and formats the incoming data, then for each antibiotic searches the metadata (2nd sheet) to locate which columns have the same concentrations, then groups the indices. The program subsequently uses those indices to group the OD_600_ readings (1st sheet) by concentration and antibiotic.

For each antibiotic, the program starts at the lowest concentration and checks if all three OD_600_ readings are above 0.15. The HT-MIC workflow automated MIC calculation incorporates the dynamic range of the microplate reader. To stay in the linear range of the spectrophotometer we conservatively set the highest OD_600_ value used as an OD_600_ = 1.00, this upper limit can be adjusted for the available equipment. Using an upper limit of 1.00 provides high confidence MIC_90_ measurements for any MIC_90_ determined, but does have the limitation of missing borderline inhibition detectable only when the actual OD_600_ value of the no drug controls are used. As we are looking for the MIC_90_, we set the OD_600_ cutoff at 10% of OD_600_ 1.00 or 0.1 plus the medium background. In our experimental setup, the OD_600_ of CA-MHB is 0.05. Adding 0.1 and 0.05 gives the value of 0.15 which is the cutoff value used to determine the MIC_90_ for the presented experimental conditions. In cases where the maximum saturation density of a culture is below an OD_600_ of 1.00 then the untreated cultures OD_600_ value should be used in place of the suggested 1.00. If desired, instead of a fixed endpoint of 0.1 OD_600_, a different cutoff value can be assigned in the code for variations in bacterial growth conditions. For example, the cutoff could be defined based on the maximum attainable OD_600_ value of the untreated control, or the value at which bacterial growth plateaus.

• If all three readings are above 0.15, it moves to check the next lowest concentration.
• If two of the readings are above 0.15, it determines if the third is at least above 0.135. If the third is above 0.135, it moves to check the next lowest concentration.
• If one of the readings is above 0.15 while two are below, it checks if that reading is below 0.165. If that reading is below 0.165, the current concentration is the MIC_90_.
• If there is disagreement with determined growth with at least one reading above 0.165 and at least one reading below 0.135 at a given antibiotic concentration, then it records the current concentration for any below 0.135 readings and moves to check the next lowest concentration for above 0.165 readings. A MIC range will be recorded representing both values.
• If none of the readings are above 0.15, then the current concentration is the MIC_90_.

After checking all the concentrations in this manner, an MIC_90_ is either determined or the MIC_90_ is labeled to be greater than the highest concentration. The program finally outputs an 8 × 2 spreadsheet as a .csv file with one column listing every antibiotic tested and the other column showing the corresponding MIC_90_. The program checks for the MIC_90_ value of each replicate from different plates and if the plates have different MIC_90_ values for each drug, it renders the results as a range, i.e., including the lowest and highest value separated by hyphen. Program run time is nearly instantaneous. Growth values of 0.135 and 0.165 are used to assess antibiotic sensitivities when growth is near the 0.15 cutoff as the HT-MIC workflow uses an average background measurement of 0.05 for absorbance from media only wells. Alternatively, a hard cutoff of 0.15 can also be used if desired.

## Result

To assess the accuracy of the high-throughput MIC prediction method we did a side-by-side comparison of the individual assigned MIC_90_ values vs. those determined by the newly developed prediction model from the HT-MIC workflow. The predicted MIC_90_ value from the HT-MIC workflow accurately identified the MIC_90_ values determined from the individual assigned method without any discrepancies (Table 1). This absolute concordance shows that the prediction model is accurate and in line with the standard method of assigning MIC_90_ values. All outputs of 1,152 wells given by microplate reader are provided in the GitHub repository under “MIC-4 strains list.xlsx” file name.

**TABLE 1:** Comparison of MIC_90_ values of microplate reader vs. software.

Strains	BAA 1605	BAA 1710	BAA 1789	AB ATCC 19606
Antibiotics				
Minocycline	2.0	1.0	2.0	0.5
Doxycycline	32.0	16.0	16.0	1.0
Tigecycline	16.0	16.0	16.0	8.0
Vancomycin	64.0	>64.0	>64.0	>64.0
Ceftriaxone	>64.0	>64.0	>64.0	32.0
Azithromycin	16.0	16.0–32.0	16.0	16.0
Meropenem	8.0	8.0	8.0	0.5
Levofloxacin	>64.0	>64.0	>64.0	1.0–2.0

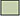
 MIC_90_ value in agreement with both techniques.

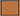
 MIC_90_ not in agreement.

MIC_90_ values in μg/mL from microplate reader and Python programming.

**FIGURE 6:**
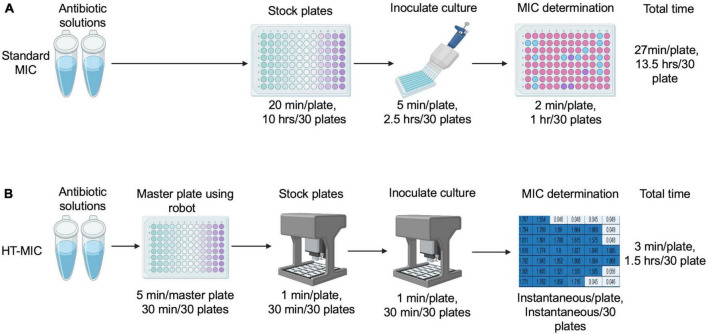
Standard MIC vs. HT-MIC time comparison. **(A)** Antibiotic stocks and stock plates are made manually and dispensed according to the CLSI guidelines. MIC determination is calculated by the user. The total time to make 30 plates is indicated. **(B)** Using HT-MIC an antibiotic master plate is created from the concentrated antibiotic stocks and dispensed to create the stock plates. Stock plates are inoculated using the OT-2 robot and MIC determination is conducted using software. The total time to make 30 plates is indicated.

Having demonstrated the accuracy of the method, we next compared the efficiency of the HT-MIC method to that of the standard MIC 96-well plate testing scheme. The HT-MIC workflow is much more efficient, and faster than the standard MIC method (Figure 6). To quantify this, we compared the amount of time needed to determine the MIC_90_ for 8 antibiotics (each examined at 11 different concentrations) in 4 bacterial strains in triplicate yielding 96 determined MIC_90_ values. Using standard MIC protocols, creating the 30 antibiotic plates needed for the MIC testing takes ∼13.5 h. Using the HT-MIC workflow, it took only ∼1.5 h to make the same set of 30 stock plates including 30 min for 5 master plates using OT-2, a savings of ∼12 h (Figure 6). Similarly, the inoculation of bacteria took ∼3 h for 30 plates when done through standard methods while the automated process accomplished it in less than 30 min (Figure 6). Moreover, the MIC_90_ prediction from automated code is instantaneous compared to the ∼1 h analyzing the readings manually. Overall, MIC determination takes ∼13.5 h to complete for 30 plates using the standard MIC method, while its takes only ∼1.5 h for the same 30 plates if done by HT-MIC method. We determined that the HT-MIC method is ∼800% faster for determining MIC values compared to standard MIC testing. Not only is the HT-MIC method faster, but it is also more efficient as it negates human pipetting error as the robot has significantly less errors.

## Discussion

The HT-MIC workflow was demonstrated to be a more efficient and accurate method for measuring MIC_90_ values when compared to standard methods. The method we used here utilizes an Opentrons OT-2 robot with a customized workflow to permit high-throughput antibiotic sensitivity experiments yielding more data with a higher degree of accuracy. The Opentrons robot’s comparatively simple setup and operation (programmable via a Python API or via a graphical user interface) allows for flexible use by non-specialized workers. Hopefully, the straightforward sharing of methods will make it simple for other labs to check and validate the work of others (Jessop-Fabre and Sonnenschein, 2019). This workflow has been developed, optimized, and previously used to screen antibiotic susceptibilities in *Staphylococcus aureus* (Dillon et al., 2019; Machado et al., 2019; Holland et al., 2022), *Pseudomonas aeruginosa* (Dillon et al., 2019; Holland et al., 2022), *Escherichia coli* (Sastry et al., 2021), and *Klebsiella pneumoniae* (Dillon et al., 2019; Holland et al., 2022) in addition to *A. baumannii* (Dillon et al., 2019; Holland et al., 2022). We have included a table (Supplementary Table 1) listing the updated susceptibility profiles of the ATCC strains from this manuscript (Ding et al., 2020; Zhu et al., 2020; Lucaßen et al., 2021; American Type Culture Collection, 2023).

The Opentrons OT-2 robot’s attractive price makes it more accessible to academic labs as it is more affordable than other automation robots available in the market (Opentrons, 2019). An additional benefit of the OT-2 system is that it includes software that is adaptable for multi-use applications. Protocols can be designed using the included software, written in python, and are also available from the open-source protocol library, which is an advantage of the OT-2 compared to other competitive liquid handling robot in the market.

The protocols used in the HT-MIC workflow are accessible through the GitHub repository provided in this paper. The protocol can be downloaded and amended for use with the Opentrons OT-2 robot to fit any specific experimental requirements. The protocol can be modified in the Opentrons protocol designer beta program allowing for the editing of steps like changing the volumes, dilution, or transferring of solution if desired.

This automated time efficient method is applicable to not only researchers in academic setting but could also be incorporated into clinical settings. Clinical microbiology labs often deal with a large influx of patient samples necessitating efficient testing processes. Using this method, sensitivity testing can be conducted with different antibiotics for multiple samples simultaneously. The automated method is hassle free and can be carried out with high accuracy. Further comparative testing against standardized clinical workflows is therefore warranted.

The protocol provided in the GitHub repository could be improved even further to decrease the experimental time and increase the data generated. Only 5 antibiotic stock plates are made from the master plate using the provided protocol. This limitation is due to the use of standard 96-well plates for the entirety of the workflow. However, efficiency could be improved through using deep well plates for the antibiotic stock plates instead of 96-well plates as larger volumes can be made. The same master antibiotic plate could then be used to make 40 antibiotic testing plates instead of 5. Using deep well plates would save even more time and will make the HT-MIC workflow even more efficient.

The link to the GitHub repository is provided here: https://github.com/suman921/HT-MIC.

## Data availability statement

The original contributions presented in this study are publicly available. This data can be found here: https://github.com/suman921/HT-MIC.

## Author contributions

ST, ON, and ND designed the project and composed the manuscript. ON created the Python MIC assignment. ST created the rest of the workflow. All authors contributed to the article and approved the submitted version.
