# Supplementary material for: Development of a high-throughput minimum inhibitory concentration (HT-MIC) testing workflow
Source: Front Microbiol. 2023 May 25;14:1079033. doi: 10.3389/fmicb.2023.1079033 (PMC10249070; doi:10.3389/fmicb.2023.1079033)
Supplement: Supplementary file 1 [file Table_1.DOCX]

**Table S1 Antibiotic susceptibility of select *Acetinobacter baumannii* strains.**

| _Strains_  _Antibiotics_ | BAA 1605 | BAA 1710 | BAA 1789 | AB ATCC 19606 |
| --- | --- | --- | --- | --- |
| Minocycline | S | S | S | S^1^ |
| Doxycycline | R | R | R | S |
| Tigecycline | S^2^ | S^2^ | S^2^ | S^1^ |
| Vancomycin | I | R | R | R |
| Ceftriaxone | R^4^ | R^2^ | R^2^ | S^3^ |
| Azithromycin | R | R | R^4^ | R |
| Meropenem | R^2^ | S^2^ | R^2^ | S^1^ |
| Levofloxacin | R^2^ | R^2^ | R^2^ | S^1^ |

R= Resistance

I= Intermediate

S= Susceptible

= Determined in this study

**References**

1. Lucaßen K, Gerson S, Xanthopoulou K, Wille J, Wille T, Seifert H, et al. Comparison of the Acinetobacter baumannii Reference Strains ATCC 17978 and ATCC 19606 in Antimicrobial Resistance Mediated by the AdeABC Efflux Pump. Antimicrob Agents Chemother. 2021;65(8):e0057021.

2. American Type Culture Collection. ATCC strain characteristics (2023). <https://www.atcc.org/> [Accessed April 18^th^, 2023].

3. Zhu Y, Lu J, Zhao J, Zhang X, Yu HH, Velkov T, et al. Complete genome sequence and genome-scale metabolic modelling of Acinetobacter baumannii type strain ATCC 19606. Int J Med Microbiol. 2020;310(3):151412.

4. Ding X, Yang C, Moreira W, Yuan P, Periaswamy B, de Sessions PF, et al. A Macromolecule Reversing Antibiotic Resistance Phenotype and Repurposing Drugs as Potent Antibiotics. Adv Sci (Weinh). 2020;7(17):2001374.
